# Gene Silencing of *Argonaute5* Negatively Affects the Establishment of the Legume-Rhizobia Symbiosis

**DOI:** 10.3390/genes8120352

**Published:** 2017-11-28

**Authors:** María del Rocio Reyero-Saavedra, Zhenzhen Qiao, María del Socorro Sánchez-Correa, M. Enrique Díaz-Pineda, Jose L. Reyes, Alejandra A. Covarrubias, Marc Libault, Oswaldo Valdés-López

**Affiliations:** 1 Laboratorio de Genómica Funcional de Leguminosas, Facultad de Estudios Superiores Iztacala, Universidad Nacional Autónoma de México, Tlalnepantla, Estado de México 54090, Mexico; maroresa@yahoo.com.mx (M.d.R.R.-S.); sscaronte@gmail.com (M.d.S.S.-C.); marioediazp96@gmail.com (M.E.D.-P.); 2 Department of Microbiology and Plant Biology, University of Oklahoma, Norman, OK 73019, USA; zhenzhen.qiao-1@ou.edu; 3 Departamento de Biología Molecular de Plantas, Instituto de Biotecnología, Universidad Nacional Autónoma de México, Cuernavaca, Morelos 62210, Mexico; jlreyes@ibt.unam.mx (J.L.R.); crobles@ibt.unam.mx (A.A.C.)

**Keywords:** common bean, soybean, Argonaute5, legume-rhizobia symbiosis

## Abstract

The establishment of the symbiosis between legumes and nitrogen-fixing rhizobia is finely regulated at the transcriptional, posttranscriptional and posttranslational levels. Argonaute5 (AGO5), a protein involved in RNA silencing, can bind both viral RNAs and microRNAs to control plant-microbe interactions and plant physiology. For instance, AGO5 regulates the systemic resistance of Arabidopsis against Potato Virus X as well as the pigmentation of soybean (*Glycine max*) seeds. Here, we show that AGO5 is also playing a central role in legume nodulation based on its preferential expression in common bean (*Phaseolus vulgaris*) and soybean roots and nodules. We also report that the expression of AGO5 is induced after 1 h of inoculation with rhizobia. Down-regulation of *AGO5* gene in *P. vulgaris* and *G. max* causes diminished root hair curling, reduces nodule formation and interferes with the induction of three critical symbiotic genes: Nuclear Factor Y-B (*NF-YB*), Nodule Inception (*NIN*) and Flotillin2 (*FLOT2*). Our findings provide evidence that the common bean and soybean *AGO5* genes play an essential role in the establishment of the symbiosis with rhizobia.

## 1. Introduction

Legumes can establish symbiosis with nitrogen-fixing bacteria (rhizobia). Through this symbiosis, atmospheric nitrogen is fixed before being assimilated (i.e., amino acids) by the plant [1]. Hence, the symbiosis between legumes and rhizobia has a considerable relevance at the ecological level. In fact, it has been estimated that the legume-rhizobia symbiosis fixes 60 million metric tons of nitrogen worldwide, and reduces the use of synthetic fertilizers [2].

To establish this symbiosis, a molecular dialog between rhizobia and legume partners is required [3,4]. This dialog begins with the detection by compatible rhizobia of legume-produced flavonoids and isoflavonoids [3,4]. In response, the rhizobia synthesize and exude lipo-chitooligosaccharides (LCOs), known as Nod Factors (NFs). The legume-host perceives NFs via LysM-domain receptor kinases Nod Factor Receptor1 and 5 (NFR1 and NFR5), both located at the legume root hair plasma membrane. Upon NFs perception, the transcription and phosphorylation of several symbiosis-related genes and proteins is activated, respectively [5]. These molecular responses are required for subsequent steps of rhizobial infection and the formation of a new organ, the nodule [4,5]. For instance, rhizobia colonize legume roots through the infection of the epidermal root hairs [6]. This colonization process begins with the attachment of rhizobia to a growing root hair tip, which induces a continuous reorientation of the tip growth, eventually leading to root hair deformation or curling [6]. In the center of this curl, an infection chamber is formed, where rhizobia are entrapped and multiply to form a micro-colony [7]. Upon rhizobia entrapment, an infection thread is formed, initiated at the location of the infection chamber and elongating to reach the nodule primordium, a meristem initiated via cell division of root cortical cells [8]. Rhizobia within the infection thread are then released into the nodule primordium cells and differentiate into bacteroids that are now able to fix nitrogen within the nodule [5].

Although the infection of root hair cells by rhizobia and the development of the nodule are biological processes controlled by two independent genetic programs, they are finely coordinated by a set of symbiotic genes [3,4]. Among these genes, *NFR5* and *NFR1*, along with *SYMRK/DMI2*/*NORK* (in *Lotus japonicus*, *Medicago truncatula* and *Medicago sativa*, respectively), which encode a leucine-rich repeat (LRR) receptor like kinase, are required for the perception and transduction of the NFs signal [4,9]. As a first response to NFs perception, legumes activate the expression of the potassium-permeable channel *DMI1*, calcium channels of the *CNGC15* family, the calcium pump *MCA8*, and nucleoporins (*NUP85*, *NUP133*, and *NENA*), as well as the expression of the mevalonate biosynthesis pathway. These genes are required to generate rapid oscillations in the nuclear and perinuclear calcium concentrations known as calcium spiking [10,11,12,13,14,15,16,17]. To decode the calcium spiking, a calcium and calmodulin-dependent protein kinase (CCaMK) is activated, which phosphorylates the transcription factor CYCLOPS [18,19]. Acting downstream, transcription factors such as Nodulation-signalling pathway1 (NSP1 and NSP2), Nodule inception protein (NIN), Ethylene response factor required for nodulation1 (ERN1), and Nuclear factor YA-1 and YB-1 (NF-YA1 and NF-YB1), are activated. The coordinated action of all these transcription factors is essential to activate the expression of different genes required for the infection of the root hair cell by rhizobia [3,4].

Recent evidence indicates that the expression of several symbiotic genes, in both rhizobia and legumes, is regulated at the epigenetic level [20]. For instance, in the legume model *M. truncatula*, demethylation of genomic DNA by DEMETER (DME) regulates the expression of genes encoding Nodule-specific Cysteine-Rich (*NCR*), Calmodulin-like, and leghemoglobin proteins, which are all required for both rhizobia differentiation and nodule development [21,22]. Similarly, the methylation pattern of the rhizobial genome affects their ability to form nodules in legumes [20]. For instance, overexpression of the DNA methyltransferase *CcrM* in *Mesorhizobium loti* leads to the deregulation of the methylation profile of the microbial genomic DNA (gDNA) leading to a delay in the development of *L. japonicus* nodules [23].

Argonaute (AGO) proteins bind small RNAs to form RNA-induced silencing complexes (RISC) involved in transcriptional and posttranscriptional gene silencing. *Arabidopsis thaliana* genome encodes ten AGO proteins (i.e., AGO1 to AGO10 [24]). Comparative genomic studies revealed the differential evolution of the *AGO* family in various flowering plants upon gene duplication and functional divergence. For instance, soybean (*Glycine max*) and common bean (*Phaseolus vulgaris*) encode 23 and 14 AGO proteins, respectively. It has been hypothesized that this duplication led to new, diverged or specific biological functions of the AGO proteins [25]. To date, AGO proteins have been involved in different developmental process and in the adaptation of plants to the changing environment, including their interaction with microbes [24]. For instance, as supported by the role of different microRNAs as major regulators of the nodule process, AGO1 has been indirectly associated with the regulation of the symbiosis between legumes and rhizobia [26]. Other AGO proteins have been involved in the control of the reproductive stage; for instance, there is evidence supporting the role of AGO5 and AGO9 in gametogenesis and in the restriction of the number of megaspore mother cells, respectively [27,28,29].

Although the genetic control underlying the establishment of the symbiosis between legumes and rhizobia has been extensively studied over the past two decades, large-scale analyses (e.g., transcriptomics and phosphoproteomics) from rhizobia-inoculated or NFs-treated roots from different legumes have revealed the existence of several potential new regulators of the symbiosis between legumes and rhizobia [30,31,32]. However, most of these genes has not been functionally characterized.

Here, we report the functional characterization of one of these potential new regulator of this symbiosis, AGO5, in common bean and soybean, two major crop legumes. Upon mining of the common bean and soybean transcriptional databases [33,34,35], we found *AGO5* preferentially expressed in roots and nodules. Further experimental validation revealed that *AGO5* is induced in response to rhizobia. To demonstrate the role of AGO5 during nodulation, we applied an RNAi strategy to down-regulate its expression. Upon silencing of *AGO5* genes in *P. vulgaris* and *G. max*, we observed a defect in nodule formation and in the induction of three critical symbiotic genes: *NF-YB*, *NIN* and Flotillin2 (*FLOT2*). Our findings show that AGO5 might play an essential role in the establishment of the symbiosis between rhizobia and legumes.

## 2. Material and Methods

### 2.1. Plant Material

Common bean (*P. vulgaris* L. cv Negro Jamapa) and soybean (*G. max* L. (Merrill) Williams 82) seeds were kindly provided by Dr. Georgina Hernandez from the Center for Genomics Science, UNAM, at Cuernavaca, Morelos, Mexico, and by Dr. Gary Stacey from the University of Missouri at Columbia, Missouri, USA. Seeds were surfaced sterilized by soaking in 70% ethanol for 1 min, followed by treatment for 10 min with 10% bleach. Seeds were subsequently washed ten-times in sterile water. Sterilized common bean seeds were germinated for two days in Petri dishes containing sterile wet germination paper under dark conditions at 25 °C. After three days of germination, common bean seedlings were transferred into 25 cm × 25 cm Petri dishes containing nitrogen-free Fähraeus medium [36] or into pots containing wet agrolite. Sterilized soybean seeds were germinated for three days in 25 cm × 25 cm Petri dishes containing nitrogen-free Fähraeus medium at 25 °C and in dark conditions. Soybean seedlings were kept under these conditions for further analyses or transferred into pots containing wet agrolite.

### 2.2. Bacterial Strains and Culture Conditions

The empty vector pTDT-DC-RNAi and the hairpin RNA interference (RNAi) construct against common bean and soybean *AGO5* (see below for details) were propagated in *Escherichia coli* DB 3.1 and DH5α cells, respectively. *E. coli* bacterial cells were handled using standard procedures.

*Agrobacterium rhizogenes* K599 strain was used to induce transgenic roots in common bean and soybean plants (see below for details). *A. rhizogenes* cells were grown on 5 mg/L peptone/3 mg/L yeast extract (PY) plates for two days at 30 °C. 100 µg/mL spectinomycin was added to select for the presence of plasmid vectors.

*Rhizobium tropici* CIAT899 and *Bradyrhizobium diazoefficiens USDA110* (reclassified from *Bradyrhizobium japonicum*) strains were used to inoculate common bean and soybean plants, respectively. *R. tropici* cells were grown on PY plates supplemented with 20 µg/mL nalidixic acid for two days at 30 °C. *B. diazoefficiens* cells were grown on YEM (0.4 g/L yeast extract, 10 g/L mannitol, 0.2 g/L MgSO_4_, 0.5 g/L KHPO_4_, 0.1g/L NaCl, pH 7.0) plates for four days at 30 °C.

### 2.3. AGO5 Down-Regulation by RNA Interference

A 150 bp 3’UTR fragment was used to generate a hairpin RNAi against *AGO5*. The amplified fragment was then cloned into the pENTR-D-TOPO (Thermo Fisher Scientific, Waltham, MA, USA) vector and verified by sequencing. The resulting pENTR-*AGO5*-RNAi plasmid was recombined into the pTDT-DC-RNAi binary vector containing the constitutively expressed fluorescent Tandem Dimer Tomato (*tdTomato*) reporter gene [37]. The correct orientation was verified by Polymerase Chain Reaction (PCR) using the primers WRKY Intron-fwd and *AGO5*-rev (for oligonucleotide sequences see Table S1). *A. rhizogenes* K599 was transformed with this RNAi vector or with the control empty vector (pTDT-DC-RNAi). *A. rhizogenes*-mediated transformation of common bean and soybean plants was performed according to [38,39], respectively. The transgenic roots were selected upon observation of TDT fluorescence with an epifluorescence stereomicroscope (SZX10, Olympus, Center Valley, PA, USA) equipped with an Olympus UC50 camera (Olympus).

### 2.4. Treatments

Three day-old soybean and common bean seedlings were transferred into nitrogen-free Fähraeus plates. Two days after transplanting, seedlings were inoculated with *R. tropici* CIAT899 (common bean symbiont) or *B. diazoefficiens* USDA110 (soybean symbiont). Inoculated seedlings were kept under dark conditions at room temperature (RT). At 1, 3, 6, 12, 24 and 48 h post inoculation, roots were harvested in liquid nitrogen and stored at −80 °C until used for transcriptional analyses. Additionally, leaves and roots from three-week-old plants as well as 25 day-old nodules were harvested in liquid nitrogen and stored at −80 °C until use. Three biological replicates were included.

Composite plants (plants with transformed root system and untransformed shoot system), expressing the construct *AGO5*-RNAi or control vector were transferred into 25 × 25 cm Petri dishes containing nitrogen-free Fähraeus medium. After four days, transgenic roots were inoculated with *R. tropici* (common bean composite plants) or *B. japonicum* (soybean composite plants). One hour after inoculation, the tdTomato fluorescent transgenic roots were harvested, then frozen in liquid nitrogen and stored at −80 °C. For this experiment seven biological replicates, each one containing roots from four different composite plants, were included.

### 2.5. Gene Expression Analysis

To analyze the expression of the *AGO5, NSP2, NIN, FLOT2*, and *ENOD40* genes, total RNA was extracted from 0.5 g of rhizobia-inoculated or mock-inoculated roots using ZR Plant RNA MiniPrep kit (Zymo Research, Irvine, CA, USA) according to manufacturer’s instructions. Genomic DNA (gDNA) was removed from purified RNA by using DNaseI RNase-free (Thermo Fisher Scientific) according to manufacturer’s instructions. 1 µg of gDNA-free total RNA was used to synthesize complementary DNA (cDNA) using Thermo Scientific RevertAid Reverse Transcriptase (Thermo Fisher Scientific) according to manufacturer’s instructions. The cDNA samples were used to analyze the expression of the above-mentioned genes by quantitative real-time PCR (qRT-PCR) in a Step-One qPCR thermocycler (Applied Biosystems, Foster, CA, USA). The housekeeping genes *PvActin* (for common bean; Phvul.008G011000.1) or *Cons6* and *Cons16* (for soybean) [40] were used to normalize gene expression levels. The expression level of different genes was calculated according to the equation E = P_eff_^(−∆Ct)^. P_eff_ is the primer set efficiency calculated using LinRegPCR program [41] and ∆ cycle threshold (Ct) was calculated by subtracting the Ct value of the housekeeping gene from the Ct values of a given gene. The nucleotide sequences of the qRT-PCR primers used in this study are provided in Table S1. For this experiment, three biological replicates were analyzed.

### 2.6. AGO5 Protein Accumulation in Response to Rhizobia

To detect the accumulation of AGO5 protein in response to rhizobia, 0.3 g of fresh rhizobia-inoculated roots (see Treatment section for details) was ground in 0.5 mL of extraction buffer (50 mM Na_4_P_2_O_7_, 1 mM Na_2_MoO_4_, 25 mM NaCl, 10 mM EDTA-Na, 0.5% PVP, 250 mM Sucrose, 50 mM HEPES, 5% glycerol, pH 7.5) supplemented with a protease inhibitor cocktail (Sigma-Aldrich, St. Louis, MO, USA). Total protein extract was centrifuged at 12,000 g for 5 min at 4 °C. Proteins were separated by 10% SDS-PAGE, and then transferred to Amersham Protan 0.2 µm nitrocellulose blotting membranes (GE Healthcare Life Sciences, Pittsburgh, PA, USA) by electroblotting. Detection of AGO5 was performed by probing membrane with anti-AGO5 antibody (Agrisera, Vännäs, Sweden; 1:1500 dilution) followed by the use of anti-IgG rabbit-HRP polyclonal antibodies (1:5000; Sigma-Aldrich). Equal loading of proteins between samples was confirmed by Coomassie blue staining. The intensity of the bands detected by western blot was quantified by densitometry using the ImageJ software [42], and the inoculated/un-inoculated ratios were obtained.

### 2.7. Root Hair Deformation Analysis

Common bean or soybean composite plants, expressing the control vector or *AGO5*-RNAi construct and growing in 25 cm × 25 cm Petri dishes containing Fahräeus medium, were inoculated with 1 mL of saturated (O.D = 1) rhizobia suspension (*R. tropici* for common bean or *B. diazoefficiens* for soybean). Forty-eight hours after inoculation, tdTomato-positive transgenic roots were collected and stained with methylene blue to maximize contrast, and then observed with a bright field microscope. A total of 15 independent biological replicates were generated, each one including ten plants.

### 2.8. Nodulation Assay

Common bean or soybean composite plants expressing the control vector or the *AGO5*-RNAi construct were transferred into pots with wet agrolite. After five-days of transplanting, common bean or soybean roots were inoculated with 3 mL of *R. tropici* or *B. diazoefficiens*, respectively. Inoculated composite plants were kept in a green house at 25–27 °C. Four weeks after inoculation, composite plants were removed from pots and those nodules developed on tdTomato-positive transgenic roots were counted. Five independent biological replicates, each one including ten plants, were generated.

### 2.9. Histology of Nodules by Light Microscopy

Images of ten whole transgenic nodules were captured using a SZX10 stereomicroscope (Olympus) equipped with an Olympus UC50 camera (Olympus). Nodule samples were sequentially dehydrated for two hours in 30%, 50%, 90% ethanol, followed by 3 treatments with 100% ethanol, with absolute ethanol-xylene (75–25%, 50–50%, 25–75%, by two hours each) and finally with 100% xylene. Upon dehydration, nodules were incubated for 24 h in xylene-paraplast (50%/50%) before embedded in LR-White resin. Semi-thin sections (25 µm) were prepared using a hand-microtome and stained with safranine in 80% ethanol. Safranine-stained semi-thin sections were examined with a NIKON ECLIPSE E200 bright-field microscope and pictures were obtained with NIS ELEMENTS BR 3.2 software (Nikon Instruments Inc., Melville, NY, USA). Representative photographs of control vector or *AGO5*-RNAi nodules are shown.

### 2.10. Sequence Collection and Phylogenetic Analysis

We performed a BLAST search to identify AGO family members in *G. max, M. truncatula*, and *P. vulgaris* based on the most recent release of their gene annotations (*Wm82.a2.v1*, *P. vulgaris v2.1* and *Mt4.0v2*). BLAST analyses were conducted using *AGO5* from soybean (*GmAGO5*) (Gm.11G190900) as a query. Potential family members were searched and validated using two BLAST resources: Phytozome and HMMER. Applying a stringent cutoff (e-value < e^−100^), we identified 10, 23, 20 and 14 *AGO* genes in Arabidopsis, soybean, *M. truncatula* and common bean genomes, respectively. The AGO proteins were validated based on the presence of the conserved Piwi and PAZ domains using Interpro bioinformatics resources [43].

In addition, to better understand the evolution of this gene family, we also included the *A. thaliana* AGO family members in our phylogenetic analysis. The phylogenetic relationships between legume and Arabidopsis *AGO* genes were established using the multiple alignment software “Molecular Evolutionary Genetic Analysis” (MEGA) [44]. Bootstrap analyses of 100 resampling replicates were made to test for the statistical significance of nodes.

### 2.11. Statistical Analyses

All the statistical analyses were conducted using R software 3.0.1 (The R project for Statistical computing). The specific statistical tests performed are indicated in the legend of the corresponding figures.

## 3. Results

### 3.1. AGO5 Is Preferentially Expressed in Roots and Nodules of Common Bean Plants

Transcriptomic analyses provide an overview of the plant transcriptional responses to any developmental and environmental stimuli. Moreover, these types of analyses also represent an excellent source to identify new potential regulators of a given biological process. In order to identify new regulators of the symbiosis between legumes and nitrogen-fixing rhizobia, we conducted a data-mining analysis on transcriptional data from *P. vulgaris* interacting with rhizobia.

**Figure 1:**
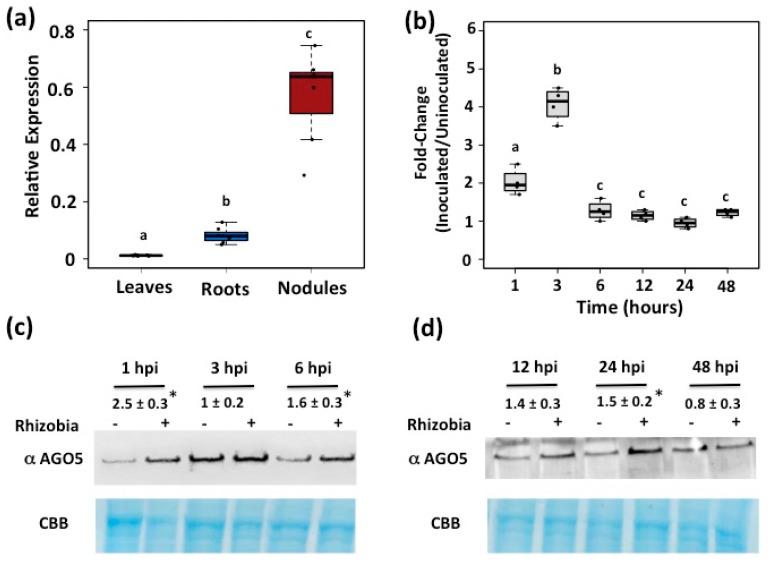
*AGO5* from *P. vulgaris* (*PvAGO5*) is preferentially expressed in root and nodules. (**a**) Expression pattern of *PvAGO5* in leaves, roots and nodules from three weeks old common bean plants; (**b**) Expression profile of *PvAGO5* in rhizobia-inoculated roots from two days old common bean plants. Box plots represent first and third quartile (horizontal box sides), minimum and maximum (outside whiskers). Data shown was obtained from four independent biological replicates. One-way ANOVA followed by a Tukey Honest Significant difference (HSD) test was performed (*p*-value < 0.01). Statistical classes sharing a letter are not significantly different. (**c**,**d**) AGO5 protein expression in rhizobia-inoculated roots from two days old common bean plants. Immunoblot shown is a representative figure from three biological replicates. The intensity of the bands was quantified densitometrically, and the inoculated/un-inoculated expression ratios were obtained for each time point. Values are mean and standard error of three biological replicates. Asterisks indicate a significant difference according to Student’s *t*-test (*p*-value < 0.01). hpi = hours post-infection.

Our data-mining analysis on the *P. vulgaris* Gene Expression Atlas [33], allowed us to identify several candidate genes, among them Phvul.011G088200.1, predicted to encode AGO5 protein. Based on available transcriptional data in common bean, this gene shows high expression in roots (including root tips) and pods, followed by nodules, leaves, and flowers (Figure S1). To validate these transcriptomic data, we evaluated the expression of this gene by qRT-PCR (Figure 1a). These quantitation analyses revealed that the Phvul.011G088200.1 gene is preferentially expressed in nodules and roots from common bean plants.

AGO5 protein from *A. thaliana* (AT2G27880; *At*AGO5) has seven domains: Argonaute *N*-terminal, Argonaute Linker1, PAZ, Argonaute Linker2, Argonaute Mid, Ribonuclease H-like, and PIWI (Figure S2). To confirm the evolutionary relationships between Phvul.011G088200.1 and *At*AGO5 proteins, we conducted a protein domain and a phylogenetic analysis (Figure S2). Comparison of *At*AGO5 and Phvul.011G088200.1 amino acid sequences showed a 60% identity between them. Furthermore, our protein domain analysis revealed that the AGO5 protein encoded in Phvul.011G088200.1 carries all the characteristic domains of AtAGO5, except the Mid domain (Figure S2). Additionally, our phylogenetic analysis showed that the protein encoded in the gene Phvul.011G088200.1 can be grouped in the *At*AGO5 clade. Altogether, these data indicate that the Phvul.011G088200.1 gene encodes for a *P. vulgaris* AGO5 (*Pv*AGO5) protein, preferentially expressed in roots and nodules of common bean plants.

### 3.2. PvAGO5 Expression Is Induced in Response to Rhizobia

Because *AGO5* is preferentially expressed in roots and nodules, we hypothesized that the expression of *AGO5* might be activated at early stages of the symbiosis between legumes and rhizobia. To test this hypothesis, we evaluated the expression of *AGO5* in common bean roots inoculated with rhizobia at various time points (1, 3, 6, 12, 24 and 48 h) (Figure 1b and Figure S3). Our expression analysis revealed that upon bacteria inoculation, *AGO5* transcript accumulates more than 2-fold during the first three hours, followed by a decrease between 6 and 48 h after bacteria inoculation (Figure 1b and Figure S3). To look at the correlation between these transcriptomic and AGO5 protein levels, we performed an immunoblotting analysis using AGO5 specific antibodies. This analysis revealed that AGO5 protein accumulation (2-fold) is detected after one hour of rhizobia inoculation, consistent with its relative transcript accumulation timing (Figure 1c). After six hours post-inoculation, a second wave of AGO5 protein accumulation was detected, this higher relative accumulation levels seems to be maintained up to 24 h after rhizobia inoculation (Figure 1d). This transcript and protein accumulation patterns indicate that AGO5 is required for both early and late stages of common bean and rhizobia symbiosis.

### 3.3. AGO5 Is Required for Rhizobia-Induced Root Hair Deformation and the Activation of Symbiosis-Specific Genes

**Figure 2:**
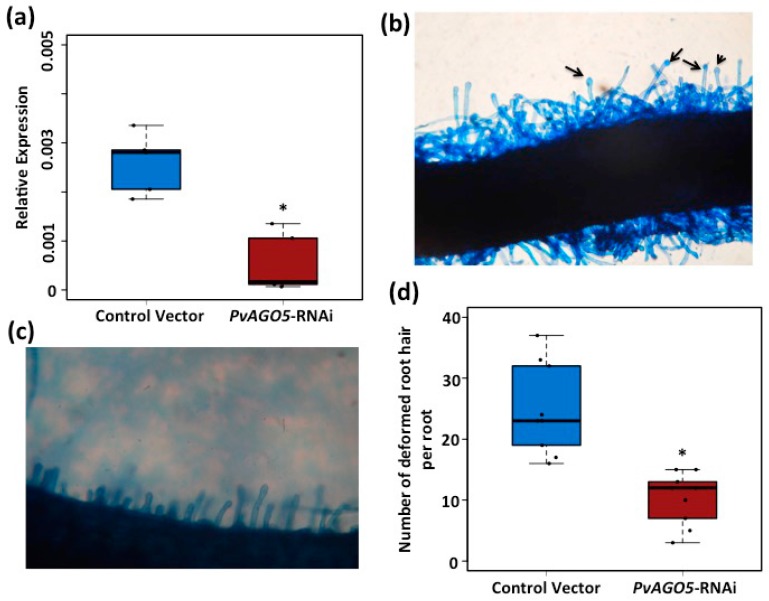
Down-regulation of *PvAGO5* reduces the rhizobia-induced root hair deformation in common bean. (**a**) *PvAGO5* expression levels in transgenic roots expressing a control vector or the *PvAGO5*-RNAi construct. Data shown was obtained from five independent biological replicates, each one containing roots from four different composite plants; (**b**) Rhizobia-induced root hair deformation in common bean transgenic roots expressing a control vector or (**c**) the *PvAGO5*-RNAi, black arrows indicate characteristic rhizobia-induced root hair deformation; (**d**) Number of rhizobia-induced root hairs observed in control transgenic roots and *PvAGO5*-silenced roots. One-way ANOVA followed by a Tukey HSD test was performed. Asterisk indicates a significant difference (*p*-value < 0.01).

Upon NFs perception by NFR5 and NFR1, different molecular and physiological responses are triggered [4], including the activation of *Early Nodulin* (*ENOD*) genes and the deformation of the root hair cell [5,45]. Because *AGO5* is expressed during the first three hours after inoculation with rhizobia, we thus hypothesized that *AGO5* might be involved in the control of some of the early steps of the symbiosis between common bean and rhizobia. To test this hypothesis, we first designed an RNAi construct targeting *PvAGO5* and utilized *A. rhizogenes*-mediated transformation to knockdown *PvAGO5*. The expression of *PvAGO5* in common bean transgenic roots expressing the RNAi construct was reduced on an average by 60% compared to roots transformed with a control vector (Figure 2a). To test whether the reduction in the expression of *PvAGO5* affects the rhizobia-induced root hair deformation, common bean transgenic roots expressing either *PvAGO5*-RNAi or control vector were inoculated with *R. tropici* CIAT899. Forty-eight hours after inoculation, 95% (*n* = 60) of the control vector-transformed roots and 20% (*n* = 60) of the *PvAGO5-RNAi*-transformed roots showed the characteristic rhizobia-induced root hair deformation (Figure 2b–d).

**Figure 3:**
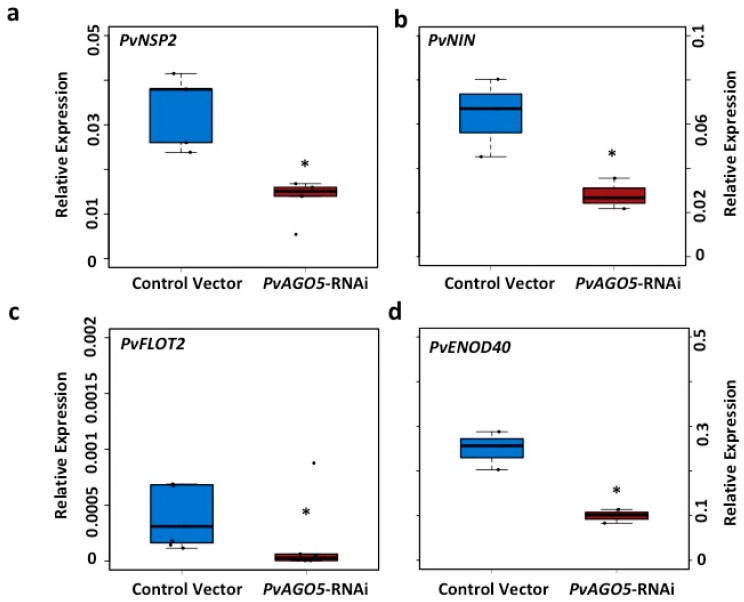
Down-regulation of *PvAGO5* affects the expression of symbiotic genes. Rhizobia-triggered expression of (**a**) Nodulation-signalling pathway2 (*PvNSP2*); (**b**) Nodule inception protein (*PvNIN*); (**c**) Flotillin2 (*PvFLOT2*) and (**d**) Early nodulin40 (*PvENOD40*) in control- and *PvAGO5*-silenced common bean transgenic roots. Data shown was obtained from six independent biological replicates, each one containing four transgenic roots from the same number of composite plants. One-way ANOVA followed by a Tukey HSD test was performed. Asterisk indicates a significant difference (*p*-value < 0.01).

The fact that *PvAGO5* is up-regulated during the first three hours following rhizobia inoculation and that the down-regulation of *PvAGO5* reduces the rate of rhizobia-induced root hair deformation, suggest that *PvAGO5* participates in promoting some the early molecular events leading to nodule development, including the transcriptional activation of *ENOD* genes. To further investigate the molecular role played by *PvAGO5*, we evaluated the expression of the symbiosis-related genes: Nodulation-signalling pathway2 (*PvNSP2*), Nodule inception protein (*PvNIN*), Flotillin2 (*PvFLOT2*), and Early nodulin40 (*PvENOD40*) in common bean transgenic roots expressing either the *PvAGO5*-RNAi construct or the control vector and inoculated for one hour with *R. tropici* (Figure 3). Our expression analysis revealed that the expression of these symbiotic genes in response to rhizobia was reduced by an average of 50% in *PvAGO5*-RNAi roots compared to the roots transformed with the control vector (Figure 3). Together, these results indicate that *PvAGO5* is involved in controlling the expression of some of the major regulatory genes, whose products participate during the early events of common bean-rhizobia symbiosis.

### 3.4. Down-Regulation of PvAGO5 Affects Nodule Development in Common Bean

**Figure 4:**
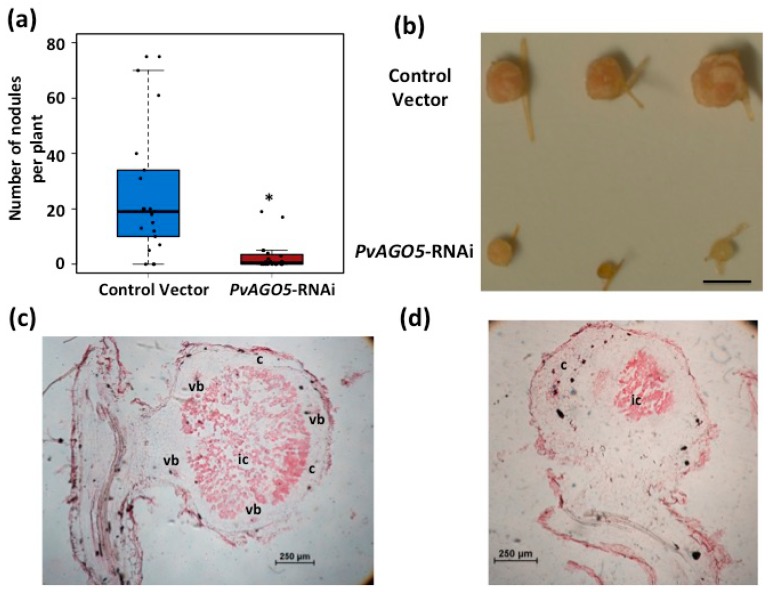
*PvAGO5*-silenced roots develop less, small and white nodules in common bean. (**a**) Nodulation assay on control- and *PvAGO5*-silenced common bean transgenic roots. Data shown was obtained from 30 independent biological replicates. One-way ANOVA followed by a Tukey HSD test was performed. Asterisk indicates a significant difference (*p*-value < 0.01); (**b**) Nodules observed in control- and *PvAGO5*-silenced common bean transgenic roots. (**c**,**d**) Safranine-stained sections of *R. tropici*-inoculated nodules showing the morphology and organization of representative samples collected from transgenic control (**c**) and *PvAGO5*-RNAi (**d**) roots. c: Cortex; ic: infected cells; vb: vascular bundle.

The relative high expression of *PvAGO5* detected in common bean mature nodules (Figure 1a) suggests that PvAGO5 might also play a role during nodule development. To test whether the down-regulation of *PvAGO5* affects the development of common bean nodules, we conducted a nodulation assay on *PvAGO5*-RNAi transgenic roots (Figure 4). Down-regulation of *PvAGO5* resulted in 60% reduction in the nodule number in silenced roots (Figure 4a). Interestingly, those nodules that reach maturity in the *PvAGO5*-silenced roots were irregular, smaller and white, in contrast to the round, large and pink nodules formed in the transgenic roots expressing the control vectors (Figure 4b and Figure S5).

To examine the structural characteristics of the nodules formed in the transgenic roots expressing *PvAGO5*-RNAi, we observed semi-thin sections of *PvAGO5*-RNAi and control vector nodules stained with safranin under a light microscope (Figure 4c,d). Control vector nodules showed the characteristic outer and inner cortexes, the nodule vascular bundles, and the central tissue that contains infected and uninfected cells (Figure 4c). In contrast, *PvAGO5*-RNAi nodules showed a clear different structure with fewer infected cells (Figure 4d). Altogether, these results indicate that the down-regulation of *PvAGO5* significantly affects common bean nodule development and rhizobia colonization.

### 3.5. AGO5 Is Also Required in Soybean to Establish Symbiosis with B. japonicum

**Figure 5:**
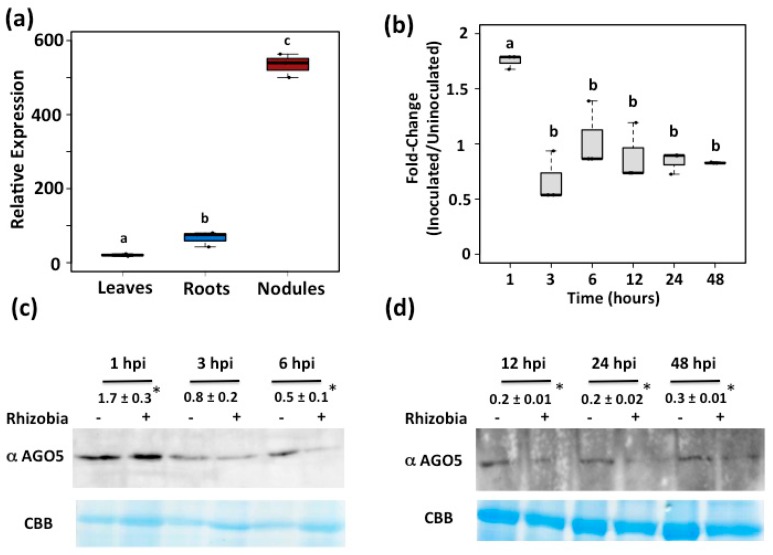
*AGO5* is preferentially expressed in soybean roots and nodules. (**a**) Expression pattern of *GmAGO5* in leaves, roots and nodules from three weeks old soybean plants; (**b**) Expression profile of *GmAGO5* in rhizobia-inoculated root from two days old soybean plants. Box plots represent first and third quartile (horizontal box sides), minimum and maximum (outside whiskers). Data shown was obtained from four independent biological replicates. One-way ANOVA followed by a Tukey HSD test was performed (*p*-value < 0.01). Statistical classes sharing a letter are not significantly different; (**c**,**d**) AGO5 protein expression in rhizobia-inoculated roots from two days old soybean plants. Immunoblot shown is a representative figure from three biological replicates. The intensity of the bands was quantified densitometrically, and the inoculated/uninoculated expression ratios were obtained for each time point. Values are mean and standard error of three biological replicates. Asterisks indicate a significant difference according to Student’s *t*-test (*p*-value < 0.01). hpi = hours post-infection.

Based on the evident effect of AGO5 on nodule development, we investigated whether this effect could be extrapolated to other legumes. For this, we examined the Soybean Knowledge Base [34,35], and found that Glyma.11g190900.1 gene encodes a putative AGO5 protein. The predicted protein AGO5 soybean protein contains the seven characteristics domains present in *At*AGO5 (Figure S2), and groups in the same clade as *At*AGO5 and *Pv*AGO5 (Figure S2). Similarly to *PvAGO5*, *GmAGO5* transcript was highly accumulated in soybean nodules and roots (Figure 5a and Figure S4), as well as in roots after one hour of *B. diazoefficiens* inoculation, this pattern was similar for *Gm*AGO5 protein accumulation level (Figure 5c,d). Despite these similarities between common and soybean, the protein levels of *Gm*AGO5 were significantly lower three hours after rhizobia inoculation in soybean. Even the second wave of AGO5 protein accumulation detected six hours after rhizobia inoculation in common bean was not observed in soybean (Figure 1d and Figure 5d). These differences in the accumulation of AGO5 proteins in response to rhizobia might be due to intrinsic differences in the way that these two legumes communicate with their symbionts.

**Figure 6:**
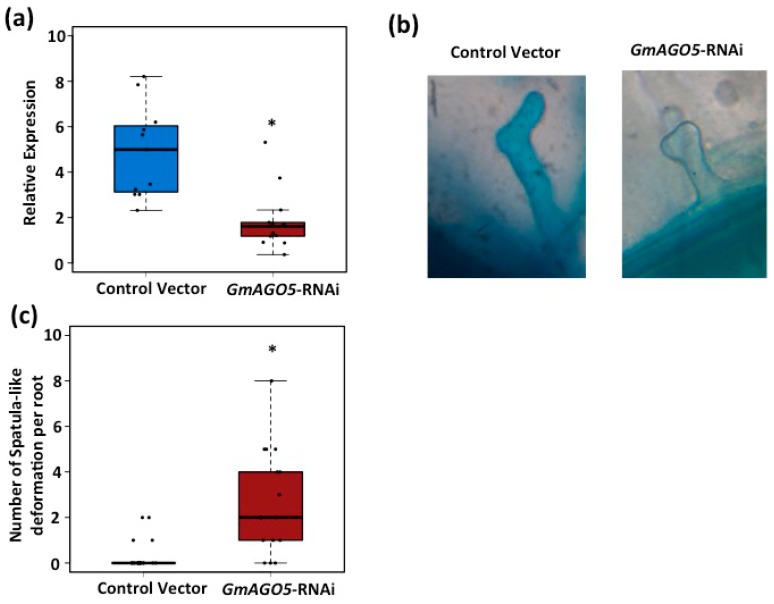
*GmAGO5*-silenced soybean roots develop rhizobia-induced spatula-like root hair deformation. (**a**) *GmAGO5* expression levels in transgenic roots expressing a control vector or the *GmAGO5*-RNAi construct. Data shown was obtained from ten independent biological replicates, each one containing roots from four different composite plants; (**b**) Rhizobia-induced root hair deformation in transgenic soybean roots expressing a control vector or the *GmAGO5*-RNAi construct; (**c**) Number of rhizobia-induced spatula-like deformed root hairs observed in control transgenic roots and *GmAGO5*-silenced roots. One-way ANOVA followed by a Tukey HSD test was performed. Asterisk indicates a significant difference (*p*-value < 0.01).

Because we observed that *GmAGO5* showed a similar expression pattern than *PvAGO5* in response to rhizobia, we also generated an *GmAGO5*-RNAi construct to silence *GmAGO5* in transgenic soybean roots produced by *A. rhizogenes*-mediated transformation. The expression of *GmAGO5* in soybean transgenic roots expressing the RNAi construct was reduced on an average by approximately 50% compared to the transcript accumulation obtained for roots transformed with a control vector (Figure 6a). To test whether the reduction in the expression of *GmAGO5* affects the typical rhizobia-induced root hair deformation, soybean transgenic roots expressing either *GmAGO5*-RNAi or control vector were inoculated with *B. diazoefficiens* USDA110. Although *GmAGO5*-RNAi transgenic roots showed characteristic rhizobia-induced root hairs, we observed that these *GmAGO5*-silenced roots predominantly exhibit “spatula-like” root hairs (Figure 6b). This root hair phenotype was observed only in *GmAGO5*-RNAi transgenic roots inoculated with *B. diazoefficiens*, indicating that this phenotype is dependent on symbiotic signaling.

**Figure 7:**
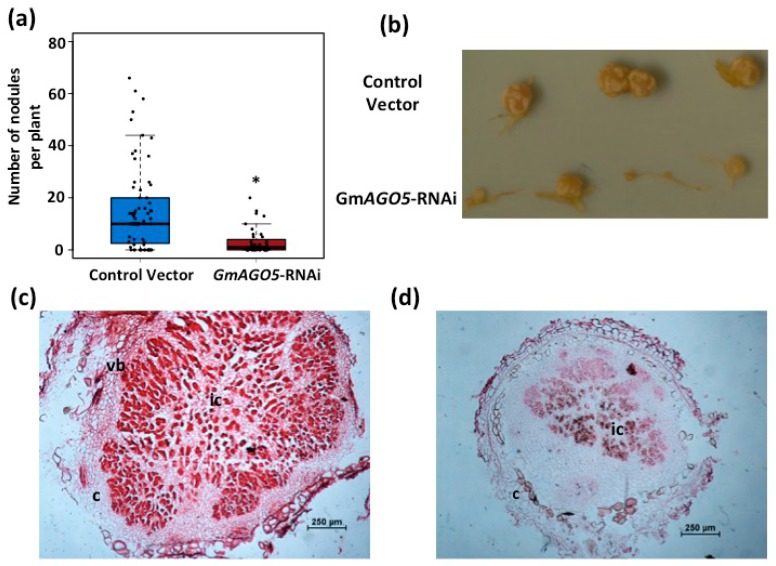
Down-regulation of AGO5 affects nodule development in soybean. (**a**) Nodulation assay on control- and *GmAGO5*-silenced common bean transgenic roots. Data shown was obtained from 30 independent biological replicates. One-way ANOVA followed by a Tukey HSD test was performed. Asterisk indicates a significant difference (*p*-value < 0.01); (**b**) Nodules observed in control- and *PvAGO5*-silenced common bean transgenic roots. (**c**,**d**) Safranine-stained sections of *B. japonicum*-inoculated nodules showing the morphology and organization of representative samples collected from transgenic control (**c**) and *GmAGO5*-RNAi (**d**) roots. c: Cortex; ic: infected cells; vb: vascular bundle.

To explore if *GmAGO5* also plays a role during nodule development similar to *PvAGO5*, we conducted a nodulation assay on soybean transgenic roots expressing *GmAGO5*-RNAi or the control vector. This assay revealed that *GmAGO5*-silenced roots developed 50% less nodules than control vector roots (Figure 7a). Similar to RNAi-*PvAGO5* nodules, the nodules formed on the *GmAGO5*-silenced transgenic roots were smaller and white, indicating a lack of leghemoglobin (Figure 7a and Figure S5). Light microscopy analysis of transgenic nodule semi-thin sections stained with safranin staining revealed that *GmAGO5*-RNAi nodules contain less infected cells than control vector-transformed nodules. These results also indicate that, similar to *PvAGO5*, silencing of *GmAGO5* results in the reduction in root hair deformation, along with reduced nodule formation efficiency and nodule morphology defects. Altogether, our data indicate that *AGO5* is playing a central role in the control of early events (i.e., expression of *NSP2*, *NIN* and *FLOT2* genes expression and rhizobia-induced root hair deformation) allowing rhizobia infection and proper development of common bean and soybean nodules.

## 4. Discussion

The symbiosis between legumes and rhizobia has been extensively studied. However, transcriptomic, proteomic and even phosphoproteomic analyses have uncovered the existence of potential new regulators of this important symbiosis [30,31,32]. Nevertheless, few of them have been functionally characterized and assigned a role in the establishment of this process [46,47,48,49]. In the present study, we provide evidence supporting the participation of AGO5 in the regulation of both early and late symbiotic processes in common bean and soybean, two major legume crops. We demonstrated that the expression of *AGO5* is induced during the first three hours of rhizobia inoculation. Further experimentation on *PvAGO5*-silenced common bean roots revealed that the rhizobia-induced root hairs deformation and the expression of *PvNSP2*, *PvNIN*, *PvFLOT2* and *PvENOD40* symbiosis-related genes were notoriously affected. Accordingly, we showed that *PvAGO5*-silenced common bean transgenic roots developed 50% less nodules and their nodules were smaller with few infected cells compared to the control transgenic roots. The effect of the down-regulation of *AGO5* in the symbiosis with rhizobia was also observed in soybean *GmAGO5*-silenced roots. These results led us to propose that AGO5 is an essential component in the establishment of the symbiosis with rhizobia in determinate legumes.

Like other AGO proteins, AGO5 binds to different types of non-coding small RNAs, particularly those initiating with cytosine, to form RISC, the complex mediating the transcriptional and posttranscriptional gene silencing [50]. The *AGO5* gene is present in most land-plants and its expression pattern is likely plant-species specific [51]. Additionally, AGO5 has been involved in the regulation of the systemic resistance of *A. thaliana* against Potato Virus X [52]. There is also evidence indicating that the *AGO5* expression is activated by different abiotic stresses, including drought and salinity in apples [51]. Recently, it was demonstrated that the soybean seed pigmentation is controlled by AGO5-associated small interference RNAs targeting the chalcone synthase transcripts [53]. Here we reported an additional AGO5 function, which might be legume-specific. However, we do not exclude the possibility that AGO5 may also play a role in the interaction of non-legume plants with soil beneficial microbes.

Early molecular responses activated upon NFs perception are critical for a successful symbiosis between legumes and rhizobia [4,5]. Some of these early responses include: protein phosphorylation [3], rapid oscillations in the nuclear and perinuclear calcium concentration (calcium spiking) [10], the synthesis and accumulation of mevalonate [17] and the activation of different *NOD* genes [3]. These early molecular responses, in turn, are finely regulated by a set of genes that altogether conform the so-called Common Symbiosis Pathway (CSP) [3,9]. One of the characteristics of the CSP participating genes is their preferential expression in roots and their early activation, few hours after NFs perception. These early molecular responses positively control root hair deformation or curling, which is required for rhizobia colonization. In this study, we showed that *PvAGO5*-silenced common bean transgenic roots showed a significant reduction in the rhizobia-induced root hair deformation. However, the deformed roots hairs were similar to those observed in control transgenic roots (Figure 1b). In contrast, *GmAGO5*-silenced soybean roots predominantly exhibited “spatula-like” root hairs (Figure 6). This spatula-like phenotype has also been observed in *M. truncatula ern1*/*ern2* and *dmi1* mutant plants [36,54]. This defect in the root hair deformation has been associated to an inhibition of the polar elongation of the root hair cell, which affects the formation of the infection chamber and the subsequent rhizobia colonization and nodule formation [36,54,55]. The fact that the *AGO5* expression is activated during the first three hours of interaction with rhizobia and that *AGO5*-silenced transgenic roots show defects in the rhizobia-induced root hair deformation, suggest that *AGO5* has a critical role in the rhizobia colonization by controlling the polar growth of root hairs and the formation of the infection chamber.

*PvAGO5*-silenced common bean transgenic roots showed 50% less accumulation of *PvNSP2*, *PvNIN*, *PvFLOT2* and *PvENOD40* symbiotic transcripts, which are required for the infection thread formation and rhizobia colonization [56,57,58,59]. NSP2 along with NSP1 forms a DNA binding complex regulating the expression of the *NIN* and *ERN1* symbiotic genes which encode transcription factors required for rhizobia infection and colonization [56]. It has been reported that *nsp2 M. truncatula* mutant plants show a reduction in rhizobia-induced root hair deformation and a complete absence of rhizobia infection [60]. In contrast, *M. truncatula nin* mutants show an excessive root hair deformation without rhizobia infection nor nodule formation [56]. Other genes required for rhizobia infection and colonization are *FLOT2* and *FLOT4* [59]. Down-regulation of these two flotillin genes seriously affects the infection thread elongation and nodule formation in *M. truncatula* transgenic roots [59]. It has also been demonstrated that the symbiotic gene *ENOD40*, which is expressed in pericycle-, nodule primordium- and nodule cells, is required for optimal nodule and bacteroid development [57]. Phenotypes similar to those reported in the *nsp2* mutant plants and *FLOTILLIN*-silenced roots were observed in the present study (Figure 3, Figure 4 and Figure 7). Considering that the down-regulation of *AGO5* significantly reduced the expression of *NSP2*, *NIN*, *FLOT2* and *ENOD40*, that the rhizobia-induced root hair deformation was significantly reduced and that the nodules formed in the *AGO5*-silenced roots were smaller and showed few infected cells, these data support our hypothesis that AGO5 is critical for rhizobia colonization. Additionally, because *AGO5*-silenced roots did not show reduction in the expression of the symbiosis-related gene *CYCLOPS* (Figure S6), but genes acting downstream of this transcription factor do (e.g., *NSP2* and *NIN*), with this data it is tempting to speculate that AGO5, along with its associated small RNAs, might act upstream of the NSP2/NSP1 complex. However, further experimentation is needed.

It has been demonstrated that both phased small interfering RNAs (phasiRNAs) and microRNAs, particularly those that with a cytosine at the 5’-end, interact with *AGO5* [50,61]. Additionally, it has also been reported that miR167 and miR172c are the most abundant microRNAs when AGO5-associated small RNAs were determined by Co-Immuno Precipitation (co-IP) assays in *A. thaliana* [50]. Interestingly, there is evidence indicating that the nodes miR172c-*APETALA2-1* and miR167-*GmARF8* control early events (e.g., rhizobia-induced root hair deformation and the activation of symbiosis-related genes) of this symbiosis and nodule development in common bean and soybean, respectively [62,63]. Hence, it is possible that the defects in the establishment of the symbiosis between common bean/soybean and rhizobia might be due to a misregulation in the activity of AGO5-dependent microRNAs that control symbiosis-related genes.

## 5. Conclusions

The data presented in this study sheds light on the role of AGO5 in the establishment of the symbiosis between legumes and rhizobia as well as the correct development of functional nodules. However, it is still not clear the role that *AGO5* is playing in this process. One possibility is that some AGO5-associated small RNAs target particular symbiotic genes. Ongoing work in our laboratory is oriented to identify the small RNAs that are associated to AGO5 in common bean and soybean under both symbiotic and non-symbiotic conditions.

## Supplementary Materials

The following are available online at www.mdpi.com/2073-4425/8/12/352/s1. Figure S1 Expression profile of *AGO5* from common bean and soybean; Figure S2 Domain and phylogenetic analysis of AGO5 from common bean and soybean; Figure S3 Expression levels of *PvAGO5* in responses to mock or rhizobia inoculation; Figure S4 Expression levels of *GmAGO5* in responses to mock or rhizobia inoculation; Figure S5 *AGO5*-RNAi nodules are small and white; Figure S6 Expression level of *PvCYCLOPs* in *AGO5*-RNAi roots; Table S1 Primers used for qRT-PCR analysis.
