# Supplementary material for: Gene Silencing of Argonaute5 Negatively Affects the Establishment of the Legume-Rhizobia Symbiosis
Source: Genes (Basel). 2017 Nov 28;8(12):352. doi: 10.3390/genes8120352 (PMC5748670; doi:10.3390/genes8120352)
Supplement: Supplementary file 1 [file genes-08-00352-s001.pdf]

Figure S1

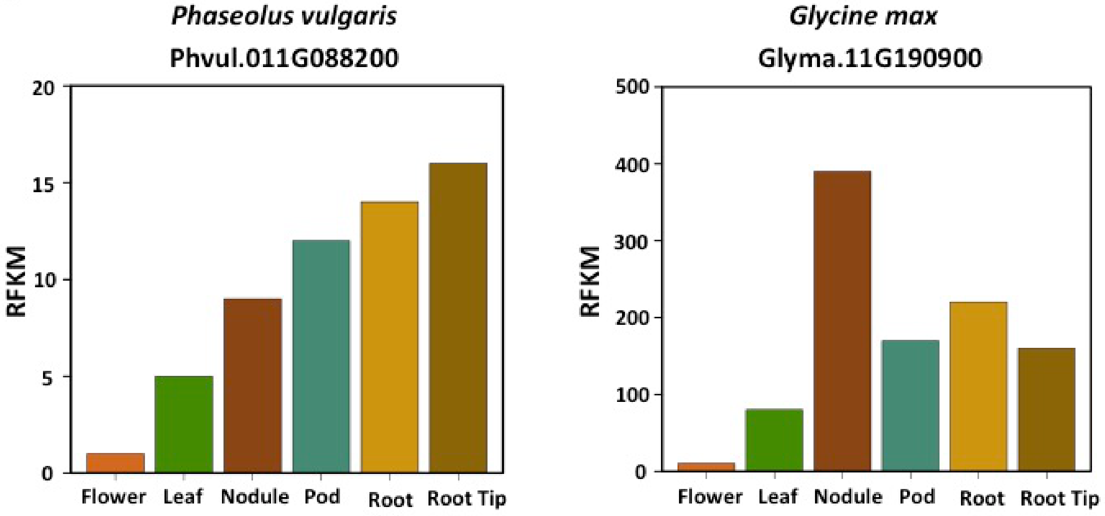

Figure S2

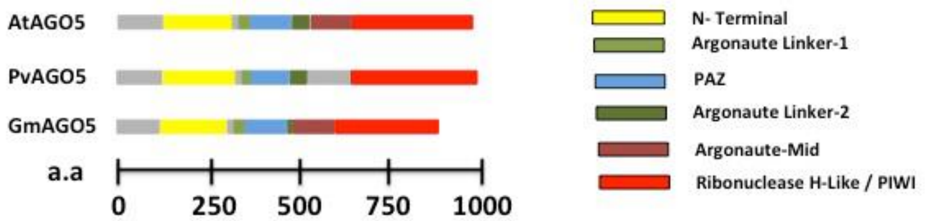

Figure S2

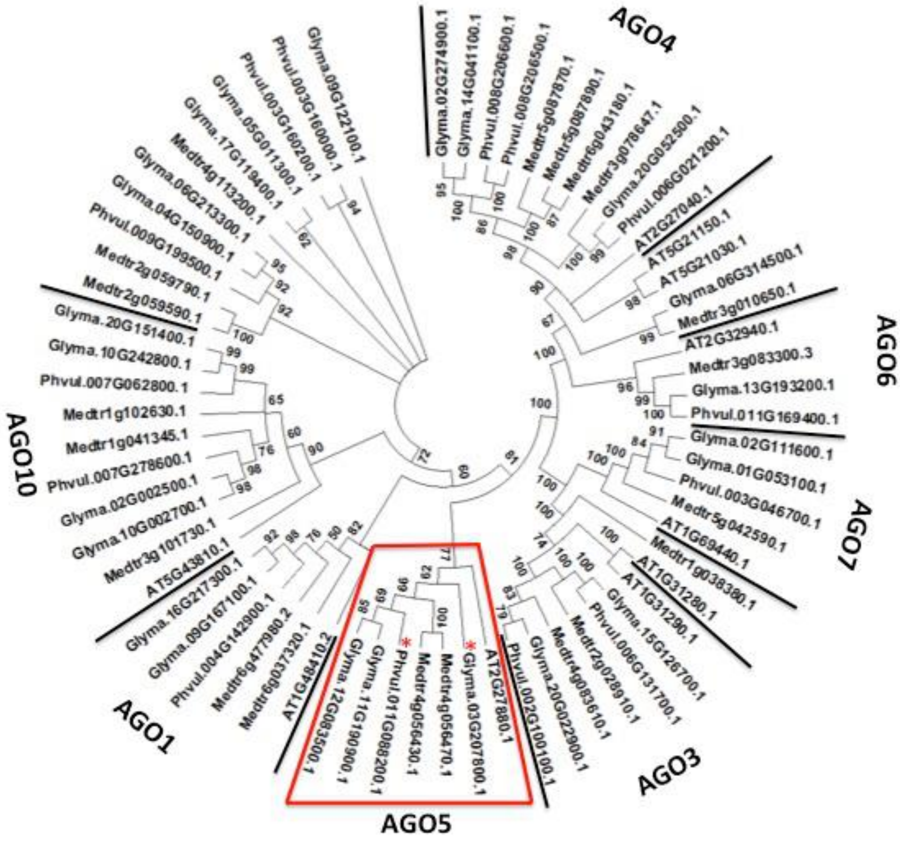

Figure S3

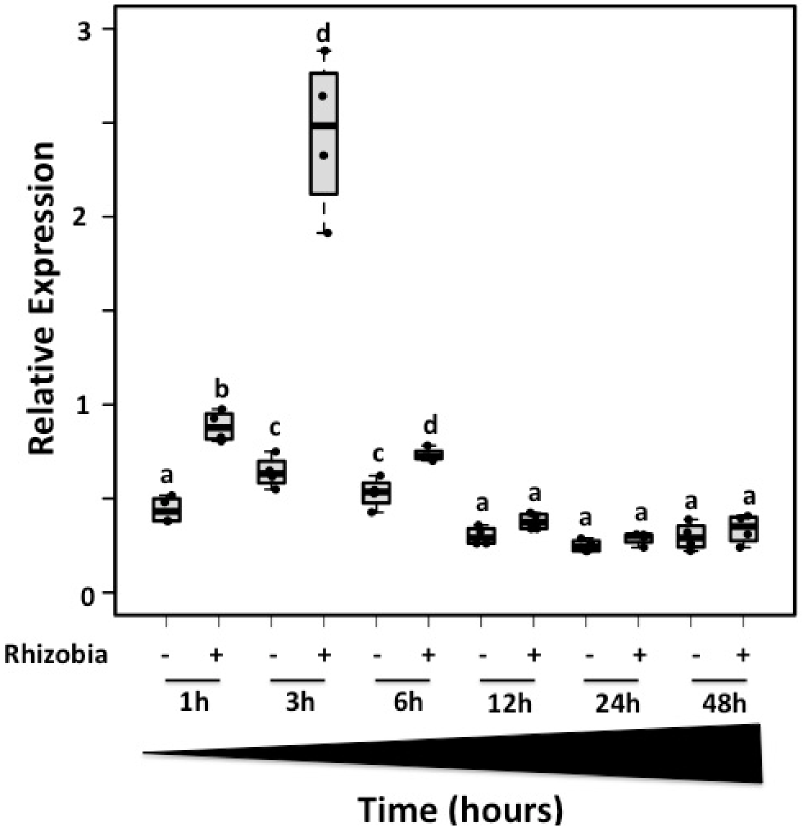

Figure S4

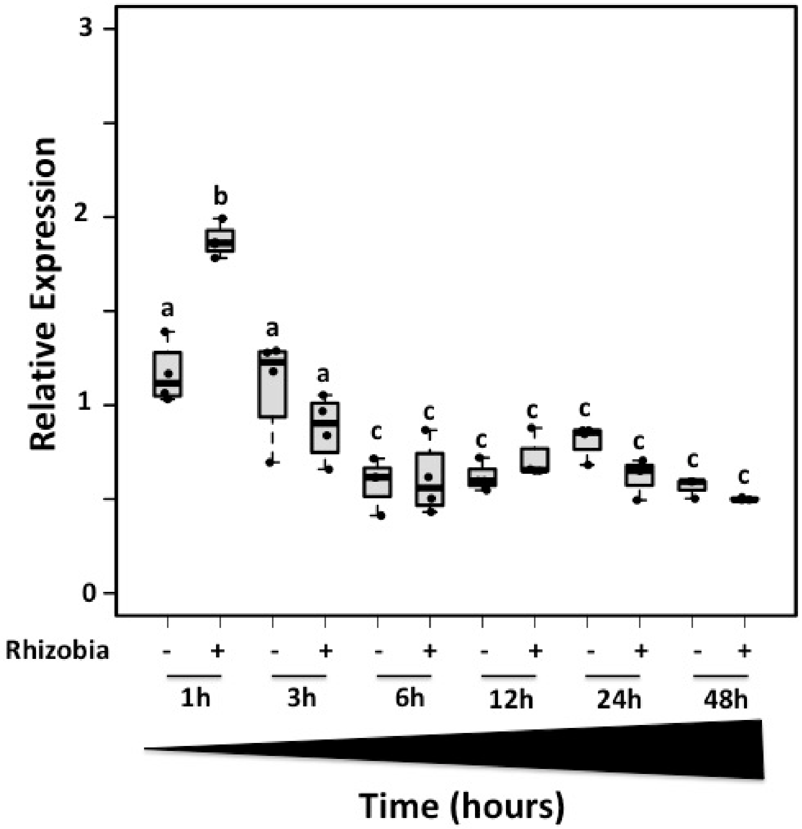

Figure S5

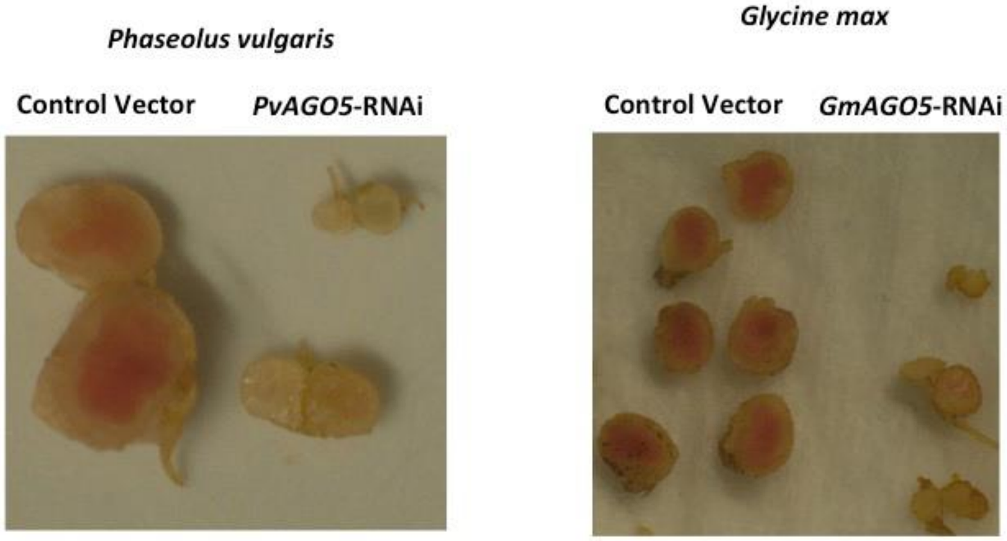

Figure S6

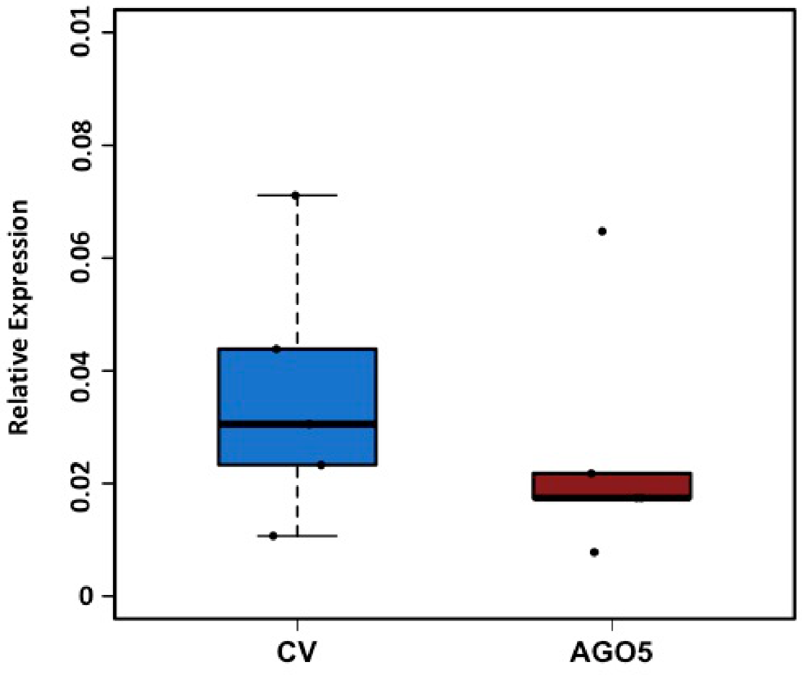

Table S1: Primers used to generate AGO5-RNAi construct and to evaluate the expression of symbiosis-related genes

Primer Name	Sequence	Purpose
PvAGO5Frw	TCA GAA TCT GGT TCT GCA TCT GGA	RNAi against PvAGO5
PvAGO5Rev	CACC TTC AAT TTC AAC TGT AGG AAA	RNAi against PvAGO5
F_GmAGO5RNAi	AAG TTC AGT TAT ATA CAA AGT TCA GTT	RNAi against GmAGO5
R_GmAGO5RNAi	CAC CCA AGC TGA CGG AAC ATT GCC TTT C	RNAi against GmAGO5
Pvago5Frw	GAG CTC GTG ATA GTA CAG ATA GAA	To check PvAGO5 gene silencing
Pvago5rev	TAG CAC AAA TTA TTG GTT AAA ATC	To check PvAGO5 gene silencing
F_GmAGO5a	AGGCTGTGGTATGCTTTTGTG	To check GmAGO5 gene silencing
R_GmAGO5a	CTCATATCAATACCCCCACCA	To check GmAGO5 gene silencing
F_PvActin	TGCAGAAGGTGAGGAGAGTTG	For qPCR data normalization in common bean
R_PvActin	GGCAGAATGAACCAGTCAAAA	For qPCR data normalization in common bean
Gm_F_cons6	AGATAGGGAAATGGTGCAGGT	For qPCR data normalization in soybean
Gm_R_cons6	CTAATGGCAATTGCAGCTCTC	For qPCR data normalization in soybean
Gm_F_cons16	TTCTGGAGTTGGAGGACACTG	For qPCR data normalization in soybean
Gm_R_cons16	GGCATCTTAACAGCAGAAGCA	For qPCR data normalization in soybean
F_PvFlot2	GGAACCCTGTCAGACAAAACA	To check the expression of PvFLOTILLIN2 by qRT-PCR
R_PvFlot2	TTCACGAATCCAAAACAACC	To check the expression of PvFLOTILLIN2 by qRT-PCR
F_PvNSP2	GACGGTTATCGGGTAGAGGAG	To check the expression of PvNSP2 by qRT-PCR
R_PvNSP2	CGGAGGAAGAAGAAGTCCAAA	To check the expression of PvNSP2 by qRT-PCR
F_PvENOD40	GGGTCCTTACCCCTCACACT	To check the expression of PvENOD40 by qRT-PCR
R_PvENOD40	TGTAGCCAAAGCCTCTCATCC	To check the expression of PvENOD40 by qRT-PCR
F_PvNIN	GGGAGAAGAGGCGTACGAAG	To check the expression of PvNIN by qRT-PCR
R_PvNIN	GTTGTGGGACACACTCCGA T	To check the expression of PvNIN by qRT-PCR
WRKY Intron-fwd	CTTCTCCAACCACAGGAATTCATC	To check RNAi orientation
